# Translating research into practice in Leeds and Bradford (TRiPLaB): a protocol for a programme of research

**DOI:** 10.1186/1748-5908-5-37

**Published:** 2010-05-21

**Authors:** Andria Hanbury, Carl Thompson, Paul M Wilson, Kate Farley, Duncan Chambers, Erica Warren, John Bibby, Russell Mannion, Ian S Watt, Simon Gilbody

**Affiliations:** 1 Department of Health Sciences, University of York, York, YO10 5DD UK; 2 Centre for Reviews and Dissemination, University of York, York, YO10 5DD, UK; 3 NHS Bradford and Airedale, Douglas Mill, Bradford, BD5 7JR, UK; 4 Health Services Management Centre, University of Birmingham, Birmingham, B15 2RT, UK

## Abstract

**Background:**

The National Institute for Health Research (NIHR) has funded nine Collaborations for Leadership in Applied Health Research and Care (CLAHRCs). Each CLAHRC is a partnership between higher education institutions (HEIs) and the NHS in nine UK regional health economies. The CLAHRC for Leeds, York, and Bradford comprises two 'research themes' and three 'implementation themes.' One of these implementation themes is Translating Research into Practice in Leeds and Bradford (TRiPLaB). TRiPLaB aims to develop, implement, and evaluate methods for inducing and sustaining the uptake of research knowledge into practice in order to improve the quality of health services for the people of Leeds and Bradford.

**Methods:**

TRiPLaB is built around a three-stage, sequential, approach using separate, longitudinal case studies conducted with collaborating NHS organisations, TRiPLaB will select robust innovations to implement, conduct a theory-informed exploration of the local context using a variety of data collection and analytic methods, and synthesise the information collected to identify the key factors influencing the uptake and adoption of targeted innovations. This synthesis will inform the development of tailored, multifaceted, interventions designed to increase the translation of research findings into practice. Mixed research methods, including time series analysis, quasi-experimental comparison, and qualitative process evaluation, will be used to evaluate the impact of the implementation strategies deployed.

**Conclusion:**

TRiPLaB is a theory-informed, systematic, mixed methods approach to developing and evaluating tailored implementation strategies aimed at increasing the translation of research-based findings into practice in one UK health economy. Through active collaboration with its local NHS, TRiPLaB aims to improve the quality of health services for the people of Leeds and Bradford and to contribute to research knowledge regarding the interaction between context and adoption behaviour in health services.

## Background

In response to the recommendation of the Chief Medical Officer's Clinical Effectiveness Group that the NHS should better utilise higher education to support initiatives to enhance the effectiveness and efficiency of clinical care [1], the National Institute for Health Research (NIHR) announced a strategy of increasing partnerships between higher education and the NHS in local health economies. One means of developing these partnerships is Collaborations in Leadership and Applied Health Research and Care or CLAHRCs. The NIHR has funded nine CLAHRCs, each with an emphasis on research that makes an impact locally and with a strong, disciplined, and strategic approach to implementing that research. The NIHR CLAHRC for Leeds, York and Bradford (LYBRA) comprises two 'research' programmes (Improving Vascular Prevention in Cardiac and Stroke Care (IMPROVE-PC), Improving the Quantity and Quality of Life in People with Addictions) and three 'implementation' programmes (Outcome Driven Stroke Care, Maternal and Child Health, and the focus of this protocol, Translating Research into Practice in Leeds and Bradford, or TRiPLaB).

The aim of TRiPLaB is to develop, implement, and evaluate methods of inducing and sustaining the uptake of research into practice in order to improve the quality of health services for the people of Leeds and Bradford. Research implementation is a complex process, highly dependent on context, and interactions between multiple, interconnected, factors at the level of individuals, groups, organisations and wider health systems [2-6]. Despite this complexity, or perhaps because of it, implementation research has often focused on individual behaviour change without reflecting on, or paying attention to, the characteristics of health technologies, the processes by which health technologies are adopted and sustained, or a workable understanding of the particular context in which implementation occurs [7].

Successive reviews of the evidence for successful translation of research findings into healthcare practice reveal that a range of implementation strategies can be successful. However, why strategies work in some circumstances but not others remains unclear [3,6,8].

Using theory to guide the exploration of the local context for implementation can help [6]. First, relevant theories enable the tailoring of strategies to the most significant barriers to translating research into practice in a given context. Second, theories enable researchers to build on existing knowledge and increase the transferability of findings to settings and contexts other than the immediate research environment [9].

TRiPLaB will use theory to guide its exploration of context in our collaborating healthcare organisations. This exploration will in turn inform the development of tailored implementation strategies for innovation delivery. The synthesis of research findings by Greenhalgh *et al*. [5] on the dissemination and implementation of research-based innovations provides the theoretical framework for TRiPLaB. Their analysis proposes that successful innovation adoption requires analysis of the characteristics of the innovation itself, the perceptions of those individuals tasked with adopting the innovation, and the wider organisational cultures in place in the setting for adoption. Shaped by diffusion of innovation theory [10], Greenhalgh *et al*. also acknowledge the influence of channels of communication, or social networks, between practitioners as important influences on whether, and how quickly, an innovation is adopted. In adopting this particular theoretical framework, TRiPLaB will explore the relative influence of these often overlooked but important elements at individual, team and organisational levels in our NHS partners [2-6]. This theory-informed exploration will form our 'diagnostic analysis' [3] of the local context in each of the NHS healthcare organisations that make up our case study series.

## Methods

Ethical approval for this study was given by York Research Ethics Committee (REC 10/H1311/1).

### The Develop, IMplement, Evaluate (DIME) approach of TRiPLaB

TRiPLaB is a multisite, longitudinal, mixed methods case study. Currently, we are working with NHS Bradford and Airedale (an NHS commissioning and community provider organisation) to translate research-based findings into practice in the areas of maternal mental health and stroke care, and with Leeds Partnership Foundation Trust (a provider of mental health services) to enhance the implementation of recent and relevant NICE guidance.

**Figure 1:**
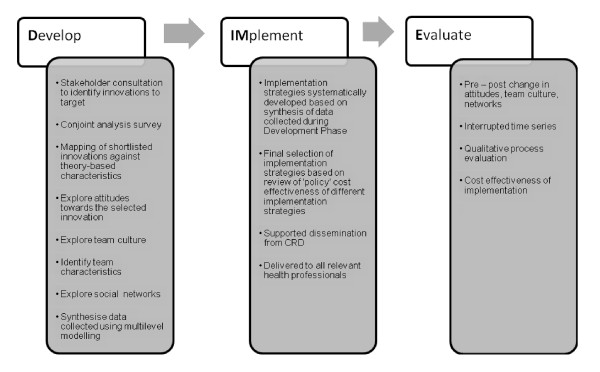
**The Develop, Implement, Evaluate model of TRiPLaB**.

Each case study will have three sequential phases (see Figure 1): the findings from the development phase (phase one) lead into the implementation phase (phase two), and the outcomes of this are assessed in the evaluation phase (phase three). The three-phase approach has been informed by the Medical Research Council's framework [11] for developing and evaluating complex interventions, acknowledges the need to use theory in planning and analysis, recognises the importance of local context, piloting, and evaluating intervention components, and the use of multiple outcome measures to evaluate intervention effectiveness. The three phases are summarised below.

Phase one is a development phase in which the innovation that is the focus of each case study will be selected and its key characteristics mapped. Theory-informed factors hypothesised as influential in health professionals' adoption of the selected innovation into routine practice are explored and mapped.

Phase two is an implementation phase in which tailored behaviour-change interventions are developed piloted and delivered using personnel from TRiPLaB and its partner organisations.

Phase Three is an evaluation phase in which changes in structure, process, and outcome are described and evaluated. We will be looking at change both within and, towards the end of the programme, between case studies.

TRiPLaB will use the resources of the Centre for Reviews and Dissemination (CRD) to increase the accessibility of research evidence to decision makers (particularly commissioners) in the NHS. Primarily, we will do this by using tailored briefings relating research evidence to specific decision problems and context in Bradford and Leeds. These 'evidence briefings' will be based on existing sources of synthesised and quality assessed evidence - for example, CRD's databases of systematic reviews (DARE) and economic evaluations (NHS EED). We will develop and implement methods for producing and disseminating evidence briefings and evaluate their perceived usefulness, costs, and use by decision makers.

### Development phase (phase one)

#### Selecting the innovation

At the start of each case study, the specific innovation to be targeted will be selected. The selection will be based on the results of: a qualitative stakeholder consultation designed to identify key topics; a conjoint analysis survey of commissioners and practitioners designed to explore those characteristics of innovations likely to influence individuals' prioritisation of them; and a mapping exercise exploring how each of the stakeholder short-listed topics 'scores' against the characteristics measured in the conjoint analysis survey (for example, local capacity and expertise for implementation, cost/impact on local budgets). We will also consider pragmatic issues, such as the presence or absence of routine data sources to aid the measurement of innovation adoption.

Stakeholder consultations will focus on identifying key topics in the relevant clinical area. For example, in NHS Bradford and Airedale, stakeholder consultation in the area of child and maternal health care with a range of commissioners and practitioners revealed the importance of maternal mental health as a focus for activity.

The conjoint survey will reveal the characteristics [6] that influence an individual's decision to prioritise one innovation over another. The factors that make up the conjoint profiles to be evaluated will include characteristics such as the strength of supporting evidence base behind an innovation and its economic costs. By conjoint analysing the characteristics of potential innovations, we will be able to 'plug in' future innovations and inform the organisation's understanding the likelihood of successful implementation. This has the obvious advantage of not having to ask the healthcare workforce or consumers to rank or rate innovations on multiple occasions. The conjoint analysis also reduces the likelihood of the TRiPLaB team targeting respondents (for example, as change agents) who may not favour the innovation eventually selected.

The mapping exercise will score short-listed innovations against characteristics measured in the conjoint analysis survey. For example, the strength of supporting evidence base for each of the short listed innovations from the stakeholder consultation will be explored through reference to published systematic reviews. The outcome of this process will be summarised in a matrix. Finally, the pragmatic factors to be considered will include whether suitable process of care and health outcome measures are available through routinely collected data to evaluate the impact of the implementation strategies, or whether tailor-made, repeatable, audits have to be established.

The selected innovation for each case study will be one that has been identified as a key topic from the stakeholder consultation that scores highly on those characteristics identified from the conjoint analysis survey as influential in commissioners' and practitioners' prioritisation of innovations, and can be monitored through tailor-made audits or, preferably, via routinely collected data. In sum, the combination of stakeholder consultation, the conjoint survey of practitioner and commissioner preferences, and the mapping exercise will enable us to select a robust but feasible innovation to target in each case site.

#### Exploring the local context

Following selection of the innovation to be targeted, we will undertake a diagnostic analysis [3] in which we will administer a second survey in each case site to measure health professionals' attitudes towards the innovation, health care team innovation culture (using the Team Climate Inventory [12]), and the social networks/communication channels between health professionals with regards to the innovation. A series of semi-structured interviews will also be conducted with a sample of the health professionals to further explore perceived barriers to implementation, and to gain a richer understanding of the influence of health care teams and social networks in the uptake and adoption of new innovations into practice.

Quantitative survey data will be synthesised using multilevel modelling (MLM) approaches to identify the hierarchical level most likely to be responsive to the implementation strategies developed. For example, if the MLM identifies healthcare team culture to be particularly influential, a multifaceted intervention specifically targeting a team's culture towards innovation might (*a priori*) be more successful than an intervention targeting only individual attitudes towards the innovation. This focus is deliberate given the current dearth of implementation research examining the influence of factors at different hierarchical levels in the health care system, and recommendations for further research in this area [13]. The qualitative data collected from the semi-structured interviews will be analysed using thematic analysis and combined with the outcome of the MLM to gain a richer understanding of the local context and to help tailor the implementation strategies.

### Implementation phase (phase two)

Development of the intervention will be systematic, specifying intervention objectives, developing specific implementation strategies to satisfy these objectives, and piloting strategies to assess their likely impact and test how they will be received by the health professionals. This piloting and modelling prior to rolling out implementation strategies/behaviour-change interventions is a necessary prerequisite stage [11]. The objectives and design of the intervention will be determined by the outcome of the development phase, in particular the results of the planned multilevel modelling. The selection and design of the actual intervention components will be informed by existing systematic, and other, reviews of the relevant literature.

Having decided on the innovation in phase one and possible implementation strategies in phase two, we will make the final choice on our implementation approach with reference to the idea of 'policy' cost effectiveness [14]. Summary data on: the innovation from Phase One (net cost per patient and likely health gain per patient); the implementation strategies under consideration (net cost of planned implementation and likely change in adoption/adherence); and local scale factors (for example, the number of NHS organisational units involved and number of patients targeted) will be combined to arrive at a policy cost effectiveness figure for each option. The combination with the highest cost effectiveness will be the option pursued.

The failure to adequately describe interventions in the context of research and the commensurate reduction in others' ability to then use successful programs -- or conversely, avoid making the same mistakes as unsuccessful ones -- is common in healthcare research [15]. For each of the case studies in the TRiPLaB program we will describe: the intervention and its component parts in sufficient detail that others could reproduce it; why the specific intervention was chosen; and a fidelity measure of how well the intervention was delivered. For example, if we undertake educational outreach or training as a component of an intervention, we will detail how many sessions each unit of analysis receive, and when and where the training took place.

### Evaluation phase (phase three)

Following the recommendation to conduct exploratory trials prior to embarking on more definitive randomised controlled trials [11], TRiPLaB will employ three different methods to evaluate the impact of the tailored implementation strategies delivered in each case study. The findings from these evaluation measures will inform (if worthwhile) later randomised controlled trials. The three methods to be used are: interrupted time series analysis of either tailor-made audit data or routinely collected data to estimate the impact of the intervention upon suitable process of care and outcome measures; comparison of pre- and post-intervention scores of survey-gathered measures of individual attitudes, team culture, and changing nature, composition, and size of social networks; and a qualitative process evaluation of why the intervention worked (or did not work).

Alongside these three primary evaluation methods we will also collect cost data on the resources used in the delivery of implementation approaches. The micro costs [16] associated with each strategy will be estimated alongside the extent of behavioural change achieved to arrive at summary estimates of implementation cost effectiveness [14] for each of the case studies in the programme.

### Interrupted time series analysis

Interrupted time series designs compare multiple 'before and after' (the introduction of a change strategy) measures to detect whether an intervention has had an effect over and above any underlying trend in the data [17]. Time series analysis has been used as a technique for evaluating the effectiveness of health care interventions [18]. In the case studies, routinely collected process (health professionals' adoption of the innovation) and health outcome measures (dependent on the innovation selected) will provide the multiple time points necessary to perform a time series analysis. Time, possible seasonal trend, and possible upward trend, commonly occurring following the introduction of a new innovation [10], will be modelled into the analysis. This will be the primary outcome measure for each case study.

### Comparison of pre- and post-intervention scores

The interrupted time series analysis will estimate the impact of the intervention upon process of care and health outcome measures; however, a comparison of pre- and post-intervention scores is also necessary to estimate whether the intervention successfully changed the factors (for example, individual attitudes, social networks and team culture) in the underlying theoretical framework that it was designed to target (based on the data synthesis through multilevel modelling in TRiPLaB's development phase in each site). This 'meditational analysis' [9] is critical when evaluating the theory used to develop change interventions, as it will inform our understanding of why an intervention either works or fails to work in the ways we intended.

### Qualitative process evaluation

Qualitative interviews with health professionals receiving the intervention will enable an exploration of their perceptions of what worked and what did not work in the intervention, providing insight into the 'black box' of intervention effectiveness [19]. In combination with the measure of fidelity taken during the implementation phase, these qualitative interviews comprise a process evaluation of the intervention, addressing recommendations to monitor intervention delivery and receipt by participants [11,20]. The data will be analysed using a framework approach [21]: familiarisation with the data, identification of a thematic framework, indexing, charting, and finally, mapping and interpretation with reference to the overall aim of TRiPLaB as well as the themes revealed by the data.

## Conclusion

TRiPLaB is a theory-informed, systematic, mixed methods approach to developing and evaluating tailored implementation strategies aimed at increasing the translation of research findings into clinical and service practice. TRiPLaB aims to play a part in improving the quality of health services for the people of Leeds and Bradford. By working alongside multiple healthcare organisations in a series of longitudinal case studies, the TRiPLaB programme will develop a richer understanding of key issues influencing the adoption of innovations in the NHS and the promotion of quality improvement in routine practice.

## Competing interests

The authors declare that they have no competing interests.

## Authors' contributions

The programme protocol was originally developed by CT, PMW, RM, ISW, JB, and SG. The protocol was further developed by AH, KF, DC, and EW. All of the authors contributed to the development and completion of the manuscript. All authors read and approved the final manuscript.
